# A case of human infection with a novel *Babesia* species in China

**DOI:** 10.1186/s40249-016-0121-1

**Published:** 2016-03-29

**Authors:** Su-Qin Man, Ke Qiao, Jie Cui, Meng Feng, Yong-Feng Fu, Xun-Jia Cheng

**Affiliations:** Department of Medical Microbiology and Parasitology, Fudan University School of Medicine, Shanghai, 200032 China; Institute of Biomedical Sciences, Fudan University, Shanghai, 200032 China

**Keywords:** Babesiosis, *Babesia* sp., Tick-borne zoonosis

## Abstract

**Background:**

Babesiosis is an uncommon but emerging tick-borne disease caused by the genus *Babesia*. In this case study, we report a case of human infection with a novel *Babesia* sp. in China.

**Findings:**

The patient in question had been suffering from repetitive occurrences of mild fever of unknown origin and fatigue for 10 years. Ring forms, tetrads, and one or two dots of chromatin or trophozoite-like organisms were observed in the patient’s thin blood smears and bone marrow smears. Using a confocal laser-scanning microscope, it was observed that the patient’s serum had reactivity with the surface proteins of the *B. microti* strain. Electron microscopy revealed oval red blood cells with 1 ~ 2 μm of knob protrusions in the cellular membrane. The results of the *Babesia*-specific nested PCR assay for 18S rRNA confirmed the presence of *Babesia* infection. The construction of a phylogenetic relationship showed clustering with *B. microti* and *B. duncani*, which was identified as a novel *Babesia* species and named as *Babesia* sp. XXB/HangZhou. Azithromycin, doxycycline, and moxifloxacin hydrochloride were shown to relieve symptoms but were not as effective after continuous usage. After atovaquone (Mepron®) administration, the patient recovered from fever and tested negative for detection of *Babesia*-specific genes.

**Conclusion:**

*Babesia* sp. XXB/HangZhou is a novel *Babesia* species, which causes mild babesiosis in an immunocompetent patient.

**Electronic supplementary material:**

The online version of this article (doi:10.1186/s40249-016-0121-1) contains supplementary material, which is available to authorized users.

## Multilingual abstract

Please see Additional file 1 for translation of the abstract into the six official working languages of the United Nations.

## Background

Babesiosis is an emerging tick-borne zoonosis in humans caused by intraerythrocytic sporozoites of the genus *Babesia*. More than 100 species of *Babesia* can infect animals, whereas only a few can infect human; primarily *B. microti* and *B. divergens*, as well as *B. venatorum*, *B. duncani*, and *Babesia* sp. MO1 [1–3]. *B. microti*, which is endemic in Northeastern and Upper Midwestern United States, generally causes mild babesiosis [4]. *B. divergens* is prevalent in Europe. A few sporadic cases of *B. microti*-like infection have also been reported in South Africa, Japan, and Taiwan.

The clinical spectrum of babesiosis ranges from an asymptomatic infection or influenza-like illness to fulminant fatal disease. The common symptoms of human babesiosis include fever, headaches, anemia, chills, myalgia, and fatigue. Severe manifestations, such as hemolysis, jaundice, thrombocytopenia, hemoglobinuria, and renal-hepatic failure, can also develop, particularly in immunocompromised patients.

In China, 75 people have been diagnosed with babesiosis until July 2015 [5]. Among them, 12 were infected with *B. microti* [6, 7], 49 with *B. venatorum* [3, 8], and two with *B. divergens* [9], while the species with which the others were infected remain unknown.

In this case study, we report a case of mild babesiosis with low-grade parasitemia caused by *Babesia* sp. infection in a human patient who has been misdiagnosed for 10 years.

## Case presentation

The patient was a 42-year-old male engineer who lives in Hangzhou city, Zhejiang province, China. He had been suffering from repetitive occurrences of mild fever of unknown origin (FUO) and fatigue for 10 years. This type of FUO is quite common, particularly in cases of chronic fatigue. In early 2005, he came down with a fever after working in the field in Jiaxing city, Zhejiang province. The patient self-reported never having a tick bite or ever receiving blood transfusion or blood products. As of late 2009, he was reassigned to an office clerk position because of his fever. Meanwhile, he was administered moxifloxacin hydrochloride and eventually recovered from the fever. However, his fatigue did not improve. He came down with a fever again and his temperature reached 38.3 °C on July 6, 2015. Administering moxifloxacin hydrochloride did not relieve his fever. That same day, he was hospitalized due to fever, generalized weakness, and fatigue.

**Fig. 1:**
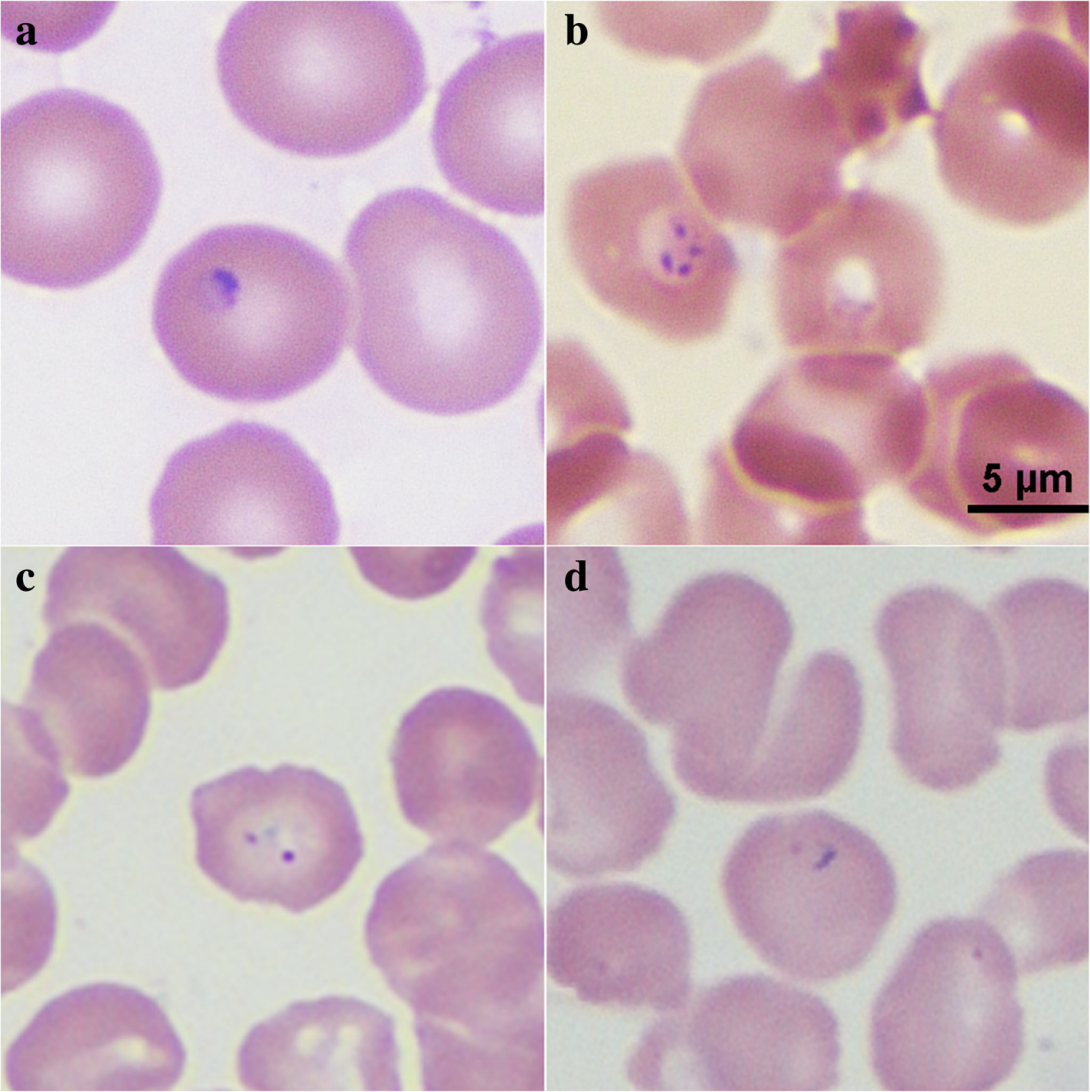
Images of erythrocytes infected with *Babesia* sp. in Giemsa-stained smears. **a** Ring form of *Babesia* sp. was observed in the patient’s thin blood smears prepared on July 25, 2015; **b** tetrads were observed in a smear of cultured blood on July 25, 2015; **c**, **d** a ring form was observed in the bone marrow smear prepared in 2005 (magnification: 100 × 10)

Blood samples obtained from the patient were sent to the laboratory to rule out parasitosis. Tetrads (Maltese cross) of *Babesia* sp. were detected using thin films of cultured blood samples obtained from the patient by Giemsa staining on July 25, 2015 (see Fig. 1). Additional forms of *Babesia* sp., such as ring and trophozoite-like forms, were also observed in smears of the patient’s blood or cultured blood samples (see Fig. 1). The small ring forms with vacuole and one or two dots of chromatin filled one third of the erythrocyte. The percentage of parasitaemia was 0.05 %.

**Fig. 2:**
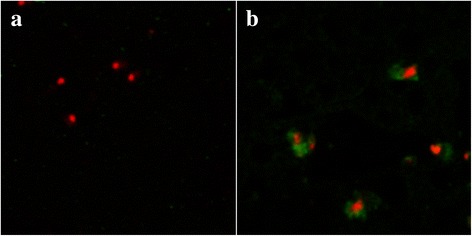
Serological reactivity of patient’s serum specimen with the antigen of *B. microti*. **a** Serum from a healthy individual was negative; **b** serum obtained from the patient on July 23, 2015 was positive (magnification: 630 × 10 × 4)

**Fig. 3:**
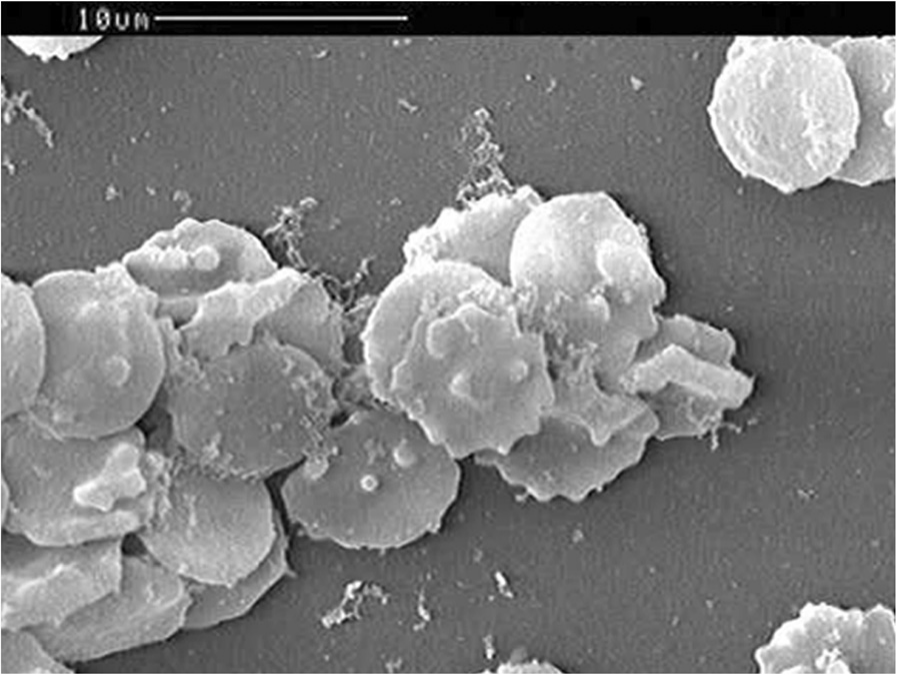
Images of erythrocytes infected with *Babesia* sp. obtained using an scanning electron microscope. Oval red blood cells with knobs, knob protrusion, or hollowness in the cellular membrane

The patient’s serum (1: 100 diluted with phosphate buffer solution (PBS)) had reactivity with the surface proteins of the *B. microti* strain by using a confocal laser-scanning microscope [10] (see Fig. 2). Electron microscopy revealed oval red blood cells with 1 ~ 2 μm of knob protrusions, or hollowness in the cellular membrane (see Fig. 3).

DNA was extracted from the patient’s blood sample on July 23, 2015. The nested polymerase chain reaction (PCR) technique was performed to amplify the partial 18S ribosomal ribonucleic acid (rRNA) gene sequence with genus-specific primers of *Babesia*. The first reaction mixture was 25 μl and contained 2 μl of DNA template, 0.5 μl of genus-specific primers (Bab 1: 5’-AAT TAC CCA ATC CTG ACA CAG G-3’ and Bab 2: 5’-TTT CGC AGT AGT TCG TCT TTA ACA-3’), 2.5 μl 10× buffer, 2.0 μl of 2.5 mM deoxynucleotide (dNTP), 1.0 μl of 50 mM magnesium sulfate (MgSO_4_), and 0.125 μl of 5 U/μl Platinum® Taq DNA Polymerase. The amplification conditions were as follows: 1) initial denaturation at 94 °C for 3 min; 2) 35 cycles of denaturation at 94 °C for 30 s, annealing at 55 °C for 30 s, and extension at 68 °C for 1 min; 3) final extension at 68 °C for 7 min. The second amplification process of nested PCR used a mixture that was 25 μl and contained 2 μl of the first nest reaction mixture, the primers (Bab 3: 5’-GAC ACA GGG AGG TAG TGA CAA GA-3’ and Bab 4: 5’-CCC AAC TGC TCC TAT TAA CCA TTA C-3’), and the same amounts of the buffer, dNTP, MgSO_4_, and Platinum® Taq DNA Polymerase as used for the first amplification reaction. The amplification conditions for the second amplification were identical to the first. The 433 base pairs of the amplicon was sequenced and shown to be most closely related to *Babesia* sp., as according to the BLAST® database (http://blast.ncbi.nlm.nih.gov/Blast.cgi).

Nested PCR was performed again based on 18S rRNA for *Plasmodium*, as previously described [11]. There were no detections of *P. falciparum*, *P. vivax*, *P. ovale*, *P. malariae*, or *P. knowlesi* in the patient’s blood.

**Fig. 4:**
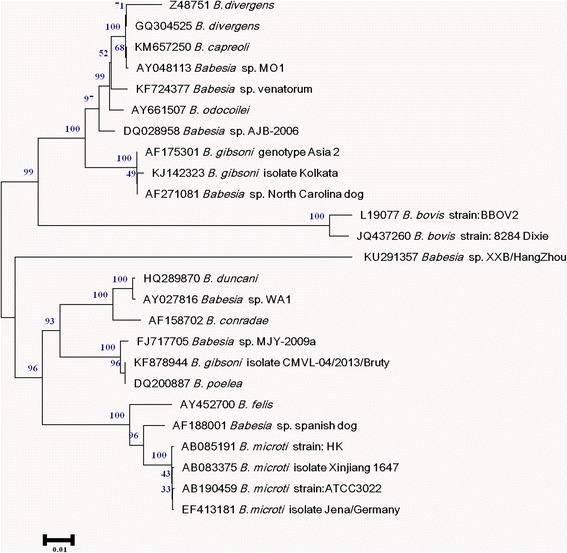
Phylogenetic tree of 18S rRNA sequences of *Babesia* sp. XXB/HangZhou. The neighbor-joining method was used to construct phylogenetic relationships using MEGA 5.05 software. The scale bar shows an evolutionary distance of 0.01 nucleotide substitution per position in the 18S rRNA sequence. Numbers near the branch are bootstrap values (1000 replicates)

To identify the species to which the isolate belonged, the whole 18S rRNA gene was amplified by nested PCR. The first nested PCR was performed with the primers RIB19 (5’-CGG GAT CCA ACC TGG TTG ATC CTG C-3’) and RIB20 (5’-CCG AAT TCC TTG TTA CGA CTT CTC-3’). The amplification conditions were as follows: 1) initial denaturation at 94 °C for 3 min; 2) 35 cycles of denaturation at 94 °C for 30 s, annealing at 55 °C for 30 s, and extension at 68 °C for 2 min; 3) final extension at 68 °C for 7 min. The second round of nested PCR was amplified with the primers Bab9 (5’-TCC ATG CTA AAA ACC TCG ACT TCG G-3’) and Bab10 (5’-TCA CCT ACG GAA ACC TTG TTA CGA CTT CTC-3’). A 1613 base pairs of the amplicon (GenBank: KU291357) was obtained, and found to be closely related to *Babesia* sp. MJY-2009a (GenBank: FJ717705 and the percentage sequence identities: 85 %). The neighbor-joining method was used to construct phylogenetic relationships of the sequence amplified from the isolate with corresponding reference sequences, using the MEGA 5.05 software (http://mega.software.informer.com/). The sequence was clustered with *B. microti* or *B. duncani* in phylogenesis (see Fig. 4). It was determined that the parasite detected from the patient’s blood belonged to a novel species of the genus *Babesia*, and was named *Babesia* sp. XXB/HangZhou.

After accurate diagnosis, atovaquone (Mepron®) was administered to the patient for 4 weeks, and there was an 1 week interval between each 2 weeks. Seven days after atovaquone administration, the patient recovered from high fever. After completion of the therapy, another round of nested PCR was performed using the genus-specific primers and DNA obtained from the patient’s blood, which showed a negative result.

Because the patient’s repetitive mild fever and fatigue have lasted for 10 years, we reviewed the Giemsa-stained bone marrow smears that were prepared in 2005. Interestingly, intraerythrocytic parasites, such as ring forms and trophozoite-like organisms, were observed in the smears. The percentage of parasitaemia was 0.01 %.

**Fig. 5:**
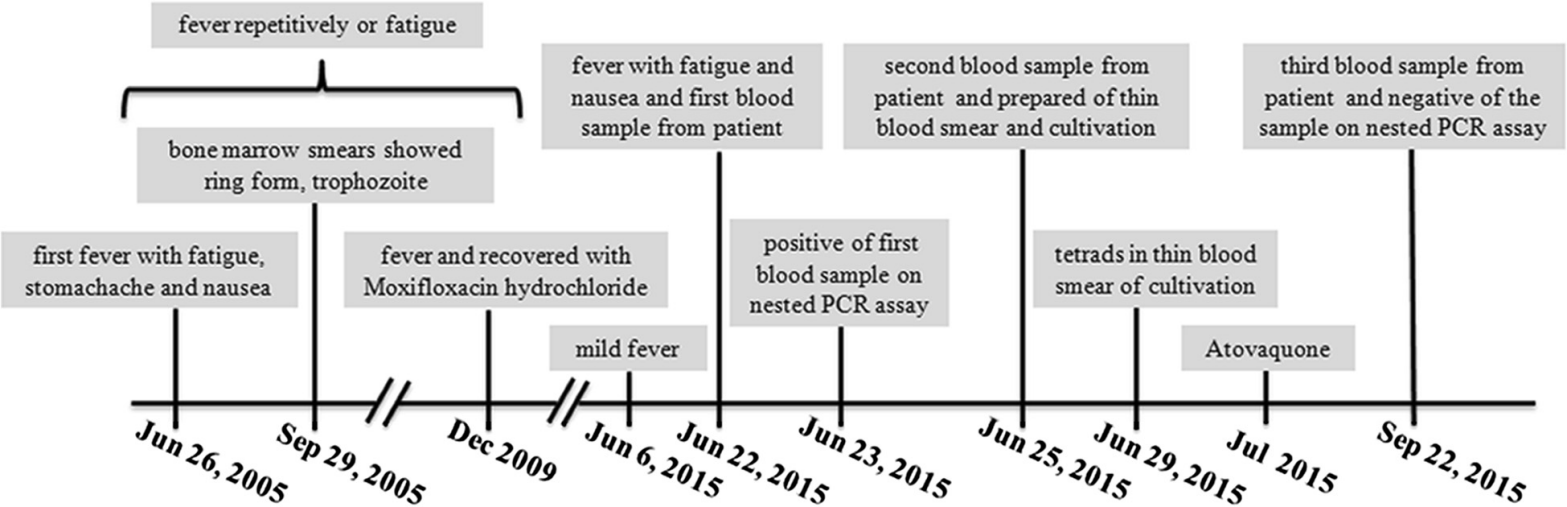
The patient’s history of the fever’s progression. The patient had been suffering from repetitive occurrences of mild fever of FUO and fatigue for 10 years

We then reviewed the patient’s history of fever progression (see Fig. 5) and found that he had sought treatment in the emergency room for fever, stomach ache, diarrhea, and nausea on July 26, 2005. Over the next 5 years, he was hospitalized several times for fever, chills, shivers, sweat, myalgia, thrombocytopenia, elevated heart rate, dysfunction of liver, and hepatosplenomegaly. Empiric treatment with an antibiotic for FUO was unresponsive. Azithromycin or doxycycline were shown to relieve his symptoms, but were not as effective after continuous usage.

Laboratory findings showed increased blood cell distribution width, erythrocyte sedimentation rate, total bilirubin, aspartate aminotransferase, alanine aminotransferase, and C-reactive protein levels. Serologic examinations, as well as observations of bone marrow, blood, urine, stool, and sputum specimens, for malaria, toxoplasmosis, human granulocytic anaplasmosis, pneumocystosis, and *Rickettsia* sp., were found to be negative.

## Conclusion

The incubation period or severity of babesiosis varies depending on the condition of the host and the species of *Babesia*. The parasite can be eradicated by the host’s immune system or the host can become an asymptomatic carrier; this is what happens in the case of human infection, i.e. what is known as an immunocompetent individual. Babesiosis can develop once host immunity declines, i.e., when the host becomes weak, undergoes splenectomy, or takes immunosuppressive drugs.

In the present case, the patient was febrile and suffered from fatigue successively for 10 years. The symptoms usually developed due to overexertion and subsided with rest. Perhaps the patient’s fatigue decreased his immunity, thus contributing to the development of babesiosis. These findings indicate that it is important to improve patients’ immunities against babesiosis.

Generally, babesiosis requires differential diagnosis to malignant malaria, as the ring-form trophozoites of *Babesia* sp. and *P. falciparum* are morphologically similar. However, the presence of the malarial pigment in the erythrocytes infected with *P. falciparum* and greater quantities of merozoites of *P. falciparum* (range from eight to 36) may help distinguish it from *Babesia* sp. In this case, we observed ring-form trophozoites and tetrads in the thin blood and bone marrow smears, which were symbolic of small *Babesia* sp.. The patient lived and worked in Zhejiang province for 10 years, which is an unstable malaria-endemic area in China [12, 13]. Hence, we also detected for *P. falciparum*, *P. vivax*, *P. ovale*, *P. malariae*, and *P. knowlesi* in the patient’s blood sample using the nested PCR technique, however, the results were negative. Furthermore, the patient had irregular occurrences of fever, but responded well to chemotherapy with anti-*Babesia* drugs.

*Babesia* sp., such as *B. microti* and *B. duncani*, can cause mild babesiosis with symptoms of fever, fatigue, anemia, or myalgia in immunocompetent patients [14]. The sequence of the isolated *Babesia* sp. XXB/HangZhou is closely related to *Babesia* sp. MJY-2009a in the BLAST® database, and belongs to the same cluster of *B. microti* and *B. duncani*. The patient had moderate fever and fatigue for ten successive years, which suggests his symptoms are of mild babesiosis. These findings indicate that *Babesia* sp. XXB/HangZhou may be the primary cause of mild babesiosis in immunocompetent patients.

Although babesiosis was defined as a notifiable disease in the United States in 2011 [15], few Chinese physicians are aware of it. With improvements in diagnostic methods and techniques, we have recently been able to detect more than ten blood specimens from patients who were suffering from FUO, with nine of them diagnosed with babesiosis. Fever is the primary clinical manifestation of babesiosis, and an indistinguishable clinical symptom between babesiosis and other forms of FUO. Babesiosis should be considered in the diagnosis of febrile patients. For the low parasitaemia in the immunocompetent patients, immunological methods and mocular methods should be applied for the detection of *Babesia*.

### Consent

The patient gave written informed consent for this case study and any accompanying images to be published. A copy of the written consent is available for review by the Editor-in-Chief of this journal.

## Additional file

Additional file 1: Multilingual abstracts in the six official working languages of the United Nations. (PDF 300 kb)

## Abbreviations

dNTP: deoxynucleotide
FUO: fever of unknown origin
MgSO_4_: magnesium sulfate
PCR: polymerase chain reaction
rRNA: ribosomal ribonucleic acid
