# Supplementary material for: A case of human infection with a novel Babesia species in China
Source: Infect Dis Poverty. 2016 Mar 29;5:28. doi: 10.1186/s40249-016-0121-1 (PMC4812642; doi:10.1186/s40249-016-0121-1)
Supplement: Additional file 1: — Multilingual abstracts in the six official working languages of the United Nations. (PDF 300 kb) [file 40249_2016_121_MOESM1_ESM.pdf]

Translation of the abstract into the six official working languages of the United Nations

حالة عدوى بشرية بنوع جديد من البابيزيا في الصين

Su-Qin Man, Ke Qiao, Jie Cui, Meng Feng, Yong-Feng Fu, Xun-Jia Cheng

موجز

معلومات عامة: داء البابيزيا (البابسيات) هو مرض غير مألوف لكنه منقول بواسطة القراد وينتج عن جنس بابيزيا. في دراسة هذه الحالة، قمنا بعمل تقرير عن حالة عدوى بشرية بنوع جديد من البابيزيا في الصين.

النتائج: لقد كان المريض موضع البحث يعاني من أعراض متكررة من الحمى الخفيفة مجهولة المصدر والإعياء منذ 10 سنوات. وقد لوحظت أشكال حلقية، ومجموعات رباعية الصبغات، وبقعة أو بقعتان من الكروماتين أو كائنات عضوية شبيهة بالأتاريف في مسحات الدم الرقيقة ومسحات نخاع العظام المأخوذة من المريض. وباستخدام مجهر متحد البؤر يستخدم الليزر للمسح الضوئي، لوحظ أن مصل دم المريض يتفاعل مع البروتينات السطحية لسلالة البابسية العكبرية. وقد أظهر الفحص المجهري الإلكتروني خلايا كرات الدم الحمراء البيضاوية بـ1~2 ميكرومتر من النتوءات العقدية في الغشاء الخلوي. وقد أكدت نتائج فحص تفاعل البلمرة التسلسلي (PCR) الخاص بالبابيزيا (البابسية) للرنا الريبوسومي (الحمض النووي الريبوزي الريبوسومي) ذي الـ18 وحدة سفيدبرج (S rRNA18) وجود العدوى البابيزيا (البابسية). وأظهر تفسير العلاقة المتعلقة بتطور السلالات وجود تعنقد في البابسية العكبرية وبابسية دونكاني، والذي اعتُبر على أنه نوع جديد من البابيزيا (البابسية) وأُطلق عليه اسم بابيزيا فصيلة إكس إكس بي/هانغتشو XXB/HangZhou. وقد تبين أن أدوية الأزيثروميسين، والدوكسيسايكلين، والموكسيفلوكساسين قد ساعدت على تخفيف الأعراض، لكنها فقدت فعاليتها بعد الاستعمال المستمر. وبعد استخدام مركب أتوفاكوين (ميبرون Mepron®)، تعافى المريض من الحمى وأظهر نتائج سلبية عند الكشف عن جينات البابسية المحددة.

الخلاصة: إن بابيزيا فصيلة إكس إكس بي/هانغتشو XXB/HangZhou هي نوع جديد من البابيزيا (البابسية)، وتتسبب في الإصابة بداء البابيزيا الخفيفة في مريض مؤهل مناعيًا.

Translated from English version into Arabic by Seham Ebied, through

TRANSLATORS
WITHOUT BORDERS

巴贝虫新种感染人体导致的巴贝虫病病例报道

满素勤，乔可，崔洁，冯萌，付永峰，程训佳

摘要

背景：巴贝虫病是由巴贝虫属原虫引起的新发蜱媒疾病。本文报道了中国发现的 1 例由巴贝虫新种引起的人体巴贝虫感染病例。

发现：显微镜观察发现 1 名近十年间歇性不明原因发热患者的外周血涂片和骨髓片的红细胞中存在有环形体、马耳他十字体、点状的染色质或不规则形的结构。扫描电子显微镜显示该患者红细胞表面具有长度为 1-2μm 的结节突起。激光共聚焦扫描显微镜发现患者血清与田鼠巴贝虫的表面特异性结合。在患者的外周血 DNA 中扩增到巴贝虫 18s rRNA 基因序列，证实该患者为巴贝虫感染。18s rRNA 序列构建的系统发生树分析结果显示该巴贝虫紧邻田鼠巴贝虫和邓肯巴贝虫的分支，为巴贝虫属的一个新种，命名为 *Babesia* sp. XXB/HangZhou。患者曾服用阿奇霉素、强力霉素或盐酸莫西沙星等药物仅可以缓解症状。经形态学、免疫学和核酸检测确诊巴贝虫感染后，给予阿托伐醌规范治疗后患者康复，外周血 DNA 巴贝虫 18s rRNA 基因 PCR 扩增未获阳性结果。

结论：对于免疫功能健全者，巴贝虫属新种 *Babesia* sp. XXB/HangZhou 可导致巴贝虫持续性温和的感染。

Translated from English version into Chinese by Fu Yongfeng and Cheng Xunjia

Un cas d’infection humaine par une nouvelle espèce de *Babesia* en Chine

Su-Qin Man, Ke Qiao, Jie Cui, Meng Feng, Yong-Feng Fu, Xun-Jia Cheng

Résumé

Contexte : La babésiose est une maladie rare mais émergente, transmise par les tiques et causée par le genre *Babesia*. La présente étude de cas rapporte un cas d’infection humaine par une nouvelle espèce de *Babesia* en Chine.

Observations : Le patient en question souffrait depuis 10 ans d’épisodes à répétition de légère fièvre de cause inconnue et de fatigue. Les frottis sanguins et médullaires de ce patient ont fait apparaître des formes annulaires, des tétrades, des points de chromatine simples et doubles ou des organismes évoquant des trophozoïtes. L’examen au microscope confocal à balayage laser a mis en évidence une réactivité du sérum du patient aux protéines de surface de la souche *B. microti*. Le microscope électronique a révélé des érythrocytes ovales, avec des saillies en bouton de 1 à 2 µm sur la membrane cellulaire. La PCR emboîtée spécifique de *Babesia* sur l’ARNr 18S a confirmé la présence d’une infection par cet organisme. L’établissement d’une relation phylogénique a montré un regroupement avec *B. microti* ou *B. duncani* et l’organisme a été identifié comme une nouvelle espèce de *Babesia* qui a été nommée *Babesia* sp. XXB/HangZhou. L’azithromycine, la doxycycline et le chlorhydrate de moxifloxacine, après avoir initialement atténué les symptômes, ont perdu en efficacité au long cours. La fièvre a disparu après l’administration d’atovaquone (Mepron®) et le patient est devenu négatif à la détection des gènes spécifiques de *Babesia*.

Conclusion : *Babesia* sp. XXB/HangZhou est une nouvelle espèce de *Babesia* qui a causé une babésiose bénigne chez un patient immunocompétent.

Translated from English version into French by Suzanne Assenat, through

TRANSLATORS WITHOUT BORDERS

Клинический случай инфицирования человека новым видом бабезиоза (*Babesia*) в Китае

Су-Цинь Ман, Кэ Цяо, Цзе Цуй, Мэн Фэн, Юн-Фэн Фу, Сюнь-Цзя Чэн

Реферат

История вопроса: Бабезиоз – это редкое, но получающее все большее распространение заболевание, передаваемое паразитами рода *Babesia*. В этом исследовании сообщается о клиническом случае инфицирования человека новым видом *Babesia* в Китае.

Результаты: Обследованный пациент на протяжении 10 лет страдал от периодически повторяющихся

приступов небольшого жара неизвестного происхождения и от усталости. В мазках крови и костного мозга пациента были обнаружены кольцевидные формы, тетрады и одна-две точки хроматина или трофозоитных организмов. С помощью растрового микроскопа с конфокальным лазером было обнаружено, что сыворотка крови пациента характеризуется реактивностью на поверхностный белок штамма *B. microti*. Электронная микроскопия выявила наличие овальных эритроцитов с утолщениями размером 1~2 мкм в клеточной мембране. Результаты гнездовой ПЦР-диагностики на наличие Babesia на присутствие видоспецифичных участков гена 18S рРНК подтвердила наличие инфицирования *Babesia*. Воссоздание филогенетических взаимоотношений показало образование кластеров с *B. microti* или *B. duncani* и было идентифицировано как новый вид *Babesia* и получило название *Babesia* sp. XXB/HangZhou. Обнаружено, что азитромицин, доксициклин и гидрохлорид моксифлоксацина облегчают симптомы заболевания, но их эффективность снижается в ходе длительного применения. После приема атоваквона (Mepron®) у пациента исчезла лихорадка, а анализы показали отрицательную реакцию на наличие генов *Babesia*.

Выводы: *Babesia* sp. XXB/HangZhou – это новый вид *Babesia*, вызывающий бабезиоз средней тяжести у пациентов, обладающих здоровым иммунитетом.

Translated from English version into Russian by Alena Hrybouskaya, through

TRANSLATORS WITHOUT BORDERS

Un caso de infección en seres humanos por una nueva especie de *Babesia* en China

Su-Qin Man, Ke Qiao, Jie Cui, Meng Feng, Yong-Feng Fu, Xun-Jia Cheng

Resumen

Antecedentes: La babesiosis es una enfermedad poco común pero emergente, transmitida por garrapatas y producida por el género *Babesia*. En este estudio de caso, reportamos un caso de infección en seres humanos por una nueva especie de *Babesia* en China.

Hallazgos: El paciente en cuestión había estado sufriendo de episodios repetitivos de fiebre leve de origen desconocido y fatiga durante 10 años. En los frotis finos de sangre del paciente y en los frotis de médula ósea se observaron formas de anillo, tétradas y uno o dos puntos de cromatina u organismos tipo trofozoítos. Mediante el uso de un microscopio confocal láser de barrido, se observó que el suero del paciente tenía reactividad con las proteínas superficiales de la cepa *B. microti*. La microscopía electrónica reveló glóbulos rojos ovalados con protuberancias de 1~2µm en forma de perilla en la membrana celular. Los resultados de los ensayos de la PCR anidada específicos para la *Babesia* para 18S ARNr confirmaron la presencia de la infección por *Babesia*. La construcción de una relación filogenética mostró agrupamientos con *B. microti* o *B. duncani,* lo que se identificó como una nueva especie de *Babesia* a la cual se denominó *Babesia* sp. XXB/HangZhou. Se observó que la azitromicina, la doxiciclina y la moxifloxacina producían alivio de los síntomas pero no eran tan efectivas luego de su uso continuado. Luego de la administración atovaquone (Mepron®) , el paciente se recuperó de la fiebre y resultó negativo para la detección de genes específicos de la *Babesia*.

Conclusión: La *Babesia* sp. XXB/HangZhou es una especie nueva de *Babesia*, que produce babesiosis leve en pacientes inmunocompetentes.

Translated from English version into Spanish by Maria Alejandra Aguada, through

TRANSLATORS
WITHOUT BORDERS
